# Placental Growth Factor, Soluble fms-Like Tyrosine Kinase 1, Soluble Endoglin, IL-6, and IL-16 as Biomarkers in Preeclampsia

**DOI:** 10.1155/2016/3027363

**Published:** 2016-10-05

**Authors:** Carmen Rădulescu, Anca Bacârea, Adina Huțanu, Rozalia Gabor, Minodora Dobreanu

**Affiliations:** ^1^Department of Obstetrics and Gynecology II, University of Medicine and Pharmacy of Tîrgu Mureș, Târgu Mureș, Romania; ^2^Department of Pathophysiology, University of Medicine and Pharmacy of Tîrgu Mureș, Târgu Mureș, Romania; ^3^CCAMF of University of Medicine and Pharmacy of Tîrgu Mureș, Târgu Mureș, Romania; ^4^Department of Laboratory Medicine, University of Medicine and Pharmacy of Tîrgu Mureș, Târgu Mureș, Romania; ^5^Department of Management and Economy, University Petru Maior of Tîrgu Mureș, Târgu Mureș, Romania

## Abstract

Preeclampsia (PE), an important cause of maternal and perinatal morbidity and mortality worldwide, is a pregnancy-related disease characterized by hypertension and proteinuria after 20 weeks of gestation. The aim of our study was to find a practical panel of biomarkers useful in early diagnosis of PE. This study was carried out at the Obstetrics and Gynecology Department in Tîrgu Mureș University Hospital, Romania, between January 2014 and July 2015 and included 68 pregnant women (47 preeclamptic women and 21 controls) with gestational age between 16 and 20 weeks at enrollment. The biomarkers PlGF, sFlt-1, sEng, IL-6, and IL-16 were determined by ELISA test. We compared the serum levels of soluble markers analysed in preeclamptic women and controls during the second and third trimesters of pregnancy and we found that the best predictor for PE was PlGF with a sensitivity of 100% at a concentration threshold of 120.16 pg/mL, a diagnosis accuracy of 70.8%, and AUC of 0.684 (*p* = 0.005). We also estimated the risk for PE according to BMI and we found that pregnant women with weight >90 kg had 7 times higher risk for PE. Second-trimester PlGF serum level may serve as an early biomarker for the diagnosis of PE.

## 1. Introduction

Preeclampsia (PE) is a pregnancy-specific disease characterized by hypertension: 140/90 mmHg or greater taken twice a day more than 4 hours apart after 20 weeks of gestation (except molar pregnancies) and proteinuria: 0.3 g or greater after a 24-hour urine sample, in previously normotensive women [1]. It is an important cause of maternal and perinatal morbidity and mortality [2, 3]. PE affects 2–8% of all pregnancies [4, 5], accounting for over 60,000 maternal deaths annually worldwide [6] The pathogenesis of PE is not completely understood. It is thought to involve defective placentation [6, 7], inadequate trophoblast invasion of maternal spiral arteries during gestation [5, 8–16], placental ischemia [5, 9, 10, 12, 14, 16], increased oxidative stress [9, 10, 16], release of antiangiogenic proteins into maternal plasma [5, 8–14, 16], excessive maternal inflammatory response [6, 8–13, 15], endothelial injury [8–10], generalized endothelial dysfunction [5, 8–10, 13, 16], hypertension [8–14, 16], and multiorganic manifestation [10, 13, 16, 17].

Vascular endothelial cells have the ability to proliferate and form a network of capillaries, process known as angiogenesis [18]. The placental vasculogenesis is dependent on several factors involved in angiogenesis and its limitation [19]. The VEGF family, VEGF-A, VEGF-B, VEGF-C, and VEGF-D, and angiopoietins are the main angiogenic factors. The limitation of angiogenesis is controlled by several antiangiogenic factors in relation to the “dialog” between hCG and extravillous trophoblast.

Placental growth factor (PlGF) is a proangiogenic protein; it peaks at 30 weeks of gestation and decreases towards term [8, 20]. It is involved in angiogenesis, vasculogenesis, and embryogenesis.

The antiangiogenic factors involved in the onset and development of PE are soluble fms-like tyrosine kinase-1 (sFlt-1) and soluble endoglin (sEng). The soluble form of vascular endothelial growth factor receptor-1 (sVEGFR-1) or soluble fms-like tyrosine kinase-1, also known as sFlt-1, binds VEGF-A, VEGF-B, and PlGF with high affinity. This leads to reduced free VEGF levels and PlGF belongs to the VEGF family and sFlt1 suppresses placental angiogenesis. The overproduction of sFlt1 due to placental hypoxia and low levels of PlGF lead to reduced free VEGF levels, thereby suppressing placental angiogenesis [21]. Another glycoprotein expressed on endothelial cells and on placental syncytiotrophoblasts is sEng, also called CD105, and is a coreceptor of TGF*β* family (transforming growth factor *β*). Endoglin decreases endothelial-nitric oxide by inhibiting TGF*β*1 and leads to endothelial dysfunction [21–23]. Hypoxia in the decidua in early phase of gestation increases the production of *α* subunit of hypoxia inducible factor 1 (HIF1*α*). It suppresses the invasion of trophoblast required for normal placenta by increasing TGF*β*3 production and increases the production of sEng in trophoblasts. sEng, a soluble receptor of TGF*β*, exacerbates hypoxia by suppressing the vasorelaxation activity of TGF*β*1. So vicious circle of placenta hypoxia occurs [12].

In hypoxic placenta, there are an increased oxidative stress, lipid peroxidation, activation of leukocytes in the intervillous space, and augmentation of endothelial expression of cytokines [24].

Interleukin-6 (IL-6) is a proinflammatory cytokine, consisting of 184 amino acids produced by various types of lymphoid and nonlymphoid cells. IL-6 plays a central role in hematopoiesis, host defense mechanism, acute phase reactions, and regulation of inflammatory and immune responses. IL-6 synthesis is regulated in part by prooxidants and antioxidants [24].

Interleukin-16 (IL-16) is a cytokine previously called lymphocyte chemoattractant factor because it could attract activated T cells. It can be synthesized by both immune and nonimmune cells, epithelial cells, mast cells, dendritic cells, monocytes, fibroblasts, B cells, and CD8+ cells [15, 25]. IL-16 can initiate and amplify an inflammatory response by releasing cell-signal molecules binding to its receptor CD4 on CD4+ T cells.

The aim of our study was to analyse the potential role of sFlt1, PlGF, sEng, IL-6, and IL-16 in predicting PE and eventually to find a practical panel of biomarkers useful in early diagnosis of preeclampsia.

## 2. Materials and Methods

This study was carried out at the Obstetrics and Gynecology Department in Tîrgu Mureș University Hospital, Romania, between January 2014 and July 2015 and included 68 pregnant women (47 preeclamptic women and 21 controls), available population with gestational age at enrollment between 16 and 20 weeks of gestation. Gestational age was calculated from the last normal menstrual period and by an early obstetric ultrasonography. Patients were followed up until delivery. The control group was selected among women without high blood pressure and proteinuria until delivery. In our study, chronic hypertension, diabetes, renal diseases, blood clotting disorders, syphilis, current infections (e.g., urinary tract infections), and chromosomal or congenital fetuses' anomalies in pregnant women were considered exclusion criteria. We also excluded hemolysed and hyperlipidemic samples.

The analysed variables are age, parity, smoking, prepregnancy body mass index (BMI), systolic blood pressure, diastolic blood pressure, proteinuria, gestational age at birth and birth weight, and serum levels of PlGF, sFlt1, sEng, IL-6, and IL-16.

The Ethics Committee of the Tîrgu Mureș University of Medicine and Pharmacy approved the study. This study included a written informed consent from each subject enrolled.

### 2.1. Methods

Maternal venous blood samples were collected between 8 and 9 o'clock in the morning. The subjects were fasted prior to blood collection and they were at bed rest. The subjects did not exercise during the study.

To determine PlGF, IL 6, and IL16 in maternal serum, we used vacutainer tubes without additives and allowed samples to clot for 30 minutes at room temperature before centrifugation for 15 minutes at 1000 ×g. In order to determine serum sFlt-1 and sEng, we put the collected blood in refrigerator at 4°C for the night; then, the blood was centrifuged for 10 minutes at 1000 ×g. We stored the samples at −80°C until assayed. The laboratory personnel performing the assays were blinded to the clinical information of each subject. The concentrations of PlGF, sFlt1, sEng, IL-6, and IL-16 were determined by enzyme-linked immunosorbent assay (ELISA): PlGF was purchased from R&D Systems Inc. (DPG00), sFlt1 (MBS2601616) and sEng (MBS269385) reagents were purchased from My BioSource, IL-6 (EK0410) and IL-16 (EK0428) were purchased from Boster Biological Technology Co., Ltd., Pleasanton, USA, and each sample was run in duplicate. The blood pressure was taken in a semirecumbent position, with a supported arm and appropriately sized cuff using a manual sphygmomanometer.

### 2.2. Statistical Analysis

Data analysis was performed using SPSS 17.0 and XLSTAT-Life 2015 for Windows 7. The data were reported as number of cases/number of patients grouped by age of patients (years), prepregnancy BMI, systolic blood pressure (SBP), diastolic blood pressure (DBP), gestational age at birth (weeks), birth weight (g), and mean and standard deviation (continuous demographic data) and as percentage for binary data. In order to compare binary data, we used Chi-square test.

In order to compare the levels of soluble markers in PE group and in controls and to assess the changes that occurred between visit 1 (second trimester of pregnancy: 16–27 weeks) and visit 2 (third trimester of pregnancy: 28–40 weeks), we used Chi-square test and Mann-Whitney *U* test. For all tests used, a *p* value < 0.05 was considered significant. The Kolmogorov-Smirnov test was used in order to evaluate the Gaussian distribution.

For calculation and graphical representation of predictive markers in relation to cut-offs values, we used, respectively, XLSTAT-Life 2015 for Windows 7 (a Demo version valid on Kovacs Computing Services, http://www.kovcomp.co.uk/support/XL-Tut/life-sensitivity-and-specificity-analysis.html) and ROC curve of each marker: AUC (area under the curve), prevalence, the cut-offs value, lower bound and upper bound for 95% confidence interval, *p* value correct classification, sensitivity (%), specificity (%), diagnosis accuracy (%), positive predictive value (PPV), negative predictive value (NPV), positive likelihood ratio (LR+), and negative likelihood ratio (LR−).

For all markers, we tested the null hypothesis (AUC is equal to 0.5) and alternative hypothesis (AUC is different from 0.5); the results were considered statistically significant when the *p* value was less than 0.05. ROC curve analysis for gestational age variable was transformed into a dichotomous variable such as 0 for gestational age lower than 34 weeks and 1 for gestational age over 34 weeks fulfilling the condition of application of this analysis method.

## 3. Results

The demographical/clinical characteristics of preeclamptic group (*n* = 47) and the control group (*n* = 21) are analysed in Table 1.

Table 2 summarizes the serum levels of soluble markers in the PE group and controls recorded in the second trimester (first hospital visit) and in the third trimester (second hospital visit).

Table 3 points out AUC, *p* value, and the prevalence of the studied markers.

The predictive characteristics of markers in relation to their cut-offs are shown in Table 4.

**Figure 1:**
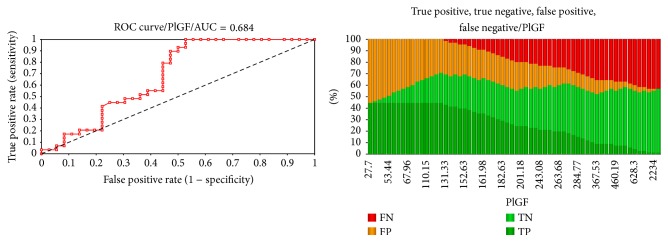
ROC curve and AUC and predictive characteristics of PlGF.

In our study, the best predictor for PE is PlGF with an AUC of 0.684 at a *p* value of 0.005 (Figure 1) with a sensitivity of 100% at a concentration threshold of 120.16 pg/mL, specificity of 47.2%, diagnostic accuracy of 70.0%, NPV of 100%, positive likelihood value (LR+) of 1.89, and negative likelihood value (LR) of 0.00.

**Figure 2:**
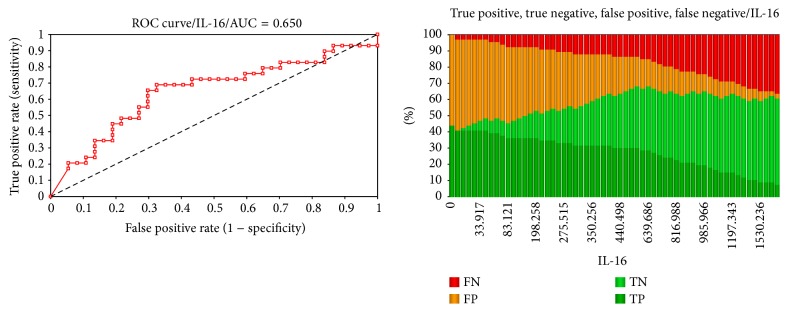
ROC curve and AUC and predictive characteristics of IL-16.

A good predictor was IL-16 with a diagnosis accuracy of 68.2% at a cut-off value of 495 pg/mL (Figure 2).

**Figure 3:**
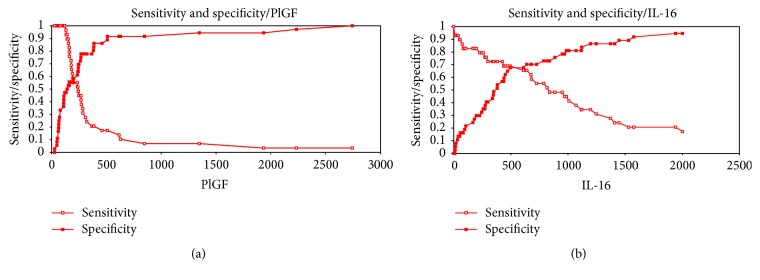
(a) Sensitivity and specificity of PlGF. (b) Sensitivity and specificity of IL-16.

Figures 3(a) and 3(b) show the sensitivity and the specificity of PlGF and IL-16 at a cut-off value of 120.16 pg/mL for PlGF and 495 pg/mL for IL-16.

The cut-off values were 2.853 ng/mL for sEng, 0.068 pg/mL for sFlt-1, 4.2 for sFlt-1/PlGF, and 2.853 pg/mL for IL-6.

The lowest predictor for PE in our study was sFlt1 with a diagnostic accuracy of 57.6%.

We have also calculated the estimated risk for developing PE in pregnant women with current weight over or under 90 kg. Patients with current weight over 90 kg have a 7 times higher risk (IC 95%: 1.73–29.77) of developing preeclampsia than those whose weight is less than 90 kg.

## 4. Discussion

Preeclampsia is a two-stage disease characterized by abnormal placentation or uteroplacental perfusion leading to increased inflammatory response and endothelial dysfunction [26, 27]. Even if the pathophysiology of PE is not fully understood, the imbalance of proangiogenic and antiangiogenic proteins seems to be a key factor in onset of this pregnancy-specific syndrome.

In our study, the best predictor for PE is PlGF. Low PlGF in pregnancy is probably due to binding of free PlGF to excessive sFlt1 secondary to a dysfunctional placenta.

Myatt et al. [27] had measured angiogenic factors in maternal plasma in the first and second trimester of pregnancy and found that maternal plasma concentration of PlGF had the best predictive power but a low sensitivity.

Kusanovic et al. [28] using a longitudinal cohort approach for maternal plasma PlGF, VEGF, sFlt1, and sEng reported that change in concentration of PlGF from first to either early or late second trimester of pregnancy was the best predictor for all PE types: mild, severe, early onset, and late onset.

In a prospective observational multicenter study from United Kingdom and Ireland, Chappell et al. [29] had studied the diagnostic accuracy of low plasma PlGF concentration in women presenting with suspected PE between 20 and 35 weeks of gestation and up to 41 weeks of gestation as a secondary analysis. The authors found that, in 287 women enrolled before 35 weeks of gestation, PlGF < 5th centile or ≤100 pg/mL had high sensitivity of 0.96 and negative predictive value of 0.98 for PE within 14 days. The specificity was lower (0.55) and AUC was 0.87.

De Vivo et al. [21] found in their study that sFlt1/PlGF ratio in maternal serum was the most accurate marker for predicting preeclampsia in both trimesters (second and third trimester), with a diagnostic accuracy of 88.5%, sensitivity of 88.5%, specificity of 88.5%, PPV of 88.5%, NPV of 88.5%, LR+ of 7.7%, and LR− of 0.13%. sFlt1 was found to be higher in the preeclamptic group in second and third trimesters but its use as predictor of PE appeared to be less useful since its diagnostic accuracy (76.9%), sensitivity (73.1%), and specificity (80.8%) were lower than PlGF, endoglin, and sFlt1/PlGF ratio. This result is probably due to the late increase of sFlt1 in preeclamptic patients. In our study, sFlt/PlGF ratio had a sensitivity of 96.6%, diagnostic accuracy of 46.2%, PPV of 40%, NVP of 100%, LR+ of 1.022, LR− of 0.621, AUC of 0.349, and *p* of 0.015.

Kumasawa et al. [30] succeeded to obtain impaired vasculogenesis, hypertension, proteinuria, and intrauterine growth restriction by genetic manipulation and expression of sFlt-1 in murine placental tissue. The authors demonstrated that excess placental sFlt1 causes impaired placentation and IUGR (intrauterine growth retardation). They concluded that although the excess sFlt1 in the placenta caused some of the preeclamptic symptoms, it is still not clear whether human preeclampsia is initiated by elevated sFlt1 or is a simply part of the end pathology.

Thadhani et al. [31] prolonged gestation in three women with preterm preeclampsia (28–30 weeks of gestation) using dextran sulphate cellulose apheresis treatment. They were able to decrease sFlt-1 levels, reduce proteinuria, and stabilize the blood pressure. Animal studies have shown that the use of angiogenic factors such as recombinant VEGF or PlGF may serve as an effective treatment to ameliorate sFlt-1 which induces endothelial damage [32, 33]. In our study, sFlt-1 in maternal serum was not a predictor for PE.

Cross et al. [34] reported symptoms resembling preeclampsia in two patients treated with bevacizumab, a recombinant, humanized, monoclonal IgG antibody that binds and inhibits VEGF. The symptoms reversed to normal after stopping bevacizumab therapy. Their study demonstrated the role of the imbalance of angiogenic and antiangiogenic factors in the pathophysiology of PE.

Foidart et al. [3] concluded in their study that the maternal plasma sEng in late PE was not significantly different from controls and therefore did not add value in screening for late PE. In our study, the diagnostic accuracy of sEng in maternal serum was of 59.1%, sensitivity 62.1%, specificity 56.8%, PPV 52.9%, NPV 65.6%, and AUC 0.610 at a *p* value of 0.106.

Unal et al. [35] found that PlGF in maternal serum was significantly lower in the second trimester of pregnancy in women who later had severe preeclampsia developed but sFlt-1 was unchanged compared with healthy pregnancies.

Thadhani et al. [36] found that low serum levels of PlGF were associated with increased risk for subsequent preeclampsia and this association was strengthened when serum levels of sFlt-1 were included in the analysis in nulliparous women.

Staff [37] studied the value of PlGF, sFlt1, and PP13 in PE from PubMed abstracts. The author highlights that PlGF, sFlt1, and PP13 seem presently to have the best predictive test values for PE, but sensitivity and specificity are still too low to be useful in a population screening setting. Staff concluded that biomarker testing should still be part of research protocols.

Takacs et al. [38] found in severe PE a significant increase of IL-6 in maternal plasma and sustained the value of this determination in the detection of PE before clinical signs appearance.

Udenze et al. [10] in their observational case control study found a significant increased levels of IL-6, TNF-*α*, and CRP (C reactive protein) in serum of women with severe PE. Similar increase of IL-6 and TNF-*α* plasma levels was also reported by Hentschke et al. [39] and Lau et al. [40]. Xie et al. [41] in a meta-analysis of TNF-*α*, IL-6, and IL-10 found high levels of these cytokines.

Kronborg et al. [42] studied plasma levels of IL-1*β*, IL-2, IL-4, IL-6, IL-8, IL-10, TNF-*α*, interferon-*γ*, and GMCSF in PE and normotensive pregnancies. The authors found that PE was associated with increased TNF-*α* between 26th and 29 weeks of pregnancy (*p* = 0.042) and increased IL-6 after 36th week (*p* = 0.004) but they concluded that measured cytokines are not eligible for predicting, monitoring, or diagnosing PE.

Ozler et al. [43] studied the value of serum levels of neopterin, TNF-*α*, and IL-6 in uncomplicated pregnant women, mild PE, severe PE, and HELLP (hemolysis, elevated liver enzymes, and low platelets) syndrome. The authors found no significant differences in the serum levels of IL-6 and TNF-*α* between the groups. Mihu et al. [9] also found elevated maternal serum IL-6 and TNF-*α* in PE women.

In our study, the diagnostic accuracy of IL-6 in maternal serum was 62.1%, with very low sensitivity (27.6%), specificity 89.2%, PPV 66.7%, NPV 61.1%, and AUC 0.568.

El-Baradie et al. [44] from Cairo studied serum concentrations of IL-15, IL-16, and beta-hCG in PE and normotensive women. For IL-16, the sensitivity was 88.89%, specificity 95.92%, PPV 88.89%, NPV 95.92%, and accuracy 94.03%. The authors found that serum IL-15 and IL-16 have a greater overall accuracy than beta-hCG in diagnosing severe PE. In our study, the accuracy of serum level of IL-16 was 68.2%, sensitivity 69%, specificity 67.6%, PPV 62.5%, NVP 73.5%, and AUC 0.650 (*p* value of 0.030).

Mbah et al. [4] found in their study that obese mothers were almost three times more likely to develop PE than women with a normal BMI. In our study, we found that pregnant women with current weight over 90 kg have a 7 times higher risk of developing preeclampsia (late onset PE) than those whose weight is less than 90 kg who have a risk of only 0.766. It is necessary to advise women at reproductive age for weight loss before becoming pregnant and not increasing in weight during pregnancy over 12.5 kg because of the risk of PE developing.

Mimura et al. [45] found in their study a possible mechanism for the protective effects of cigarette smoking against PE, thus proposing a therapeutic potential of nicotine or other nicotinic acetylcholine receptor agonists for PE. Nicotine restores proangiogenic functions to endothelial cells pretreated with sFlt1 and/or sEng.

Oggè et al. [46] found different concentration levels of angiogenic and antiangiogenic factors in maternal plasma and serum. In women with preeclampsia, the median concentrations of sVEGFR-1, sVEGFR-2, PlGF, and sEng were significantly higher in serum than in plasma. They did not find a significant difference between serum and plasma concentration for PlGF and sEng between the second and the third trimester.

Delivery of the fetus and placenta remains the most effective treatment in severe preeclampsia. Prophylactic use of aspirin, vitamins, fish oil, and calcium has limited value in PE.

The fact that the study was conducted in one region of the country, with a relatively small number of participants and a sample of convenience, is a limitation of the study.

## 5. Conclusion

In our study, the best predictor for PE is PlGF. Second-trimester PlGF serum levels may serve as an early biomarker for the diagnosis of onset preeclampsia over a 120.16 pg/mL threshold.

## Figures and Tables

**Table 1:** Clinical characteristics of the two groups of women: preeclamptic and control.

	Preeclamptic group *n* = 47	Control group *n* = 21	*p* value
Age (years)	29 ± 5.4	27 ± 6.6	0.097^*∗*^
Nulliparas (%)	77	76	0.996^*∗*^
Smoker (%)	25.5	28.6	0.507^*∗*^
Prepregnancy BMI	27.3 ± 6.8	21.1 ± 2.8	0.019^*∗∗*^
Systolic blood pressure (mmHg)	152 ± 17	114 ± 9	0.000^*∗*^
Diastolic blood pressure (mmHg)	97 ± 11	70 ± 5	0.000^*∗*^
Proteinuria (%)	100	0	NA
GA at birth (weeks)	37 ± 1.2	38.7 ± 2.2	0.006^*∗∗*^
Birthweight (g)	3117 ± 644	3345 ± 372	0.000^*∗∗*^

^*∗*^Chi-square test.

^*∗∗*^Mann–Whitney *U* test.

**Table 2:** Serum levels of soluble markers analysed in PE and controls.

	Second trimester (first visit)			Third trimester (second visit)		
	PE group *n* = 47	CTRL group *n* = 21	*p* value	PE group *n* = 47	CTRL group *n* = 21	*p* value
sEng (ng/mL)	2.78 (.34–5.11)	3.52 (.97–6.15)	0.058^*∗∗*^	2.75 (.67–6.65)	3.12 (1.04–5.47)	0.000^*∗∗*^
sFlt1 (pg/mL)	310.22 (9–8077)	142.05 (.009–.67)	0.000^*∗*^	514.23 (1–13557.0)	201.32 (.00–1.17)	0.000^*∗*^
PlGF (pg/mL)	319.31 (27.7–2234.0)	383.07 (21.21–2744.00)	0.001^*∗∗*^	313.36 (45.50–1559.2)	696.93 (127.22–2123.90)	0.001^*∗∗*^
sFlt1/PlGF	3.35 (.02–125.45)	.86 (.05–3.78)	0.000^*∗∗*^	9.62 (.000–297.91)	.51 (.00–4.20)	0.000^*∗∗*^
IL-6 (pg/mL)	2.62 (.01–58.83)	1.64 (.02–14.42)	0.000^*∗*^	4.57 (.00–76.36)	.01 (.00–.02)	0.000^*∗*^
IL-16 (pg/mL)	698.26 (.00–2001.0)	810.67 (20.80–2001.0)	0.018^*∗∗*^	882.91 (0.90–2001.0)	1186.72 (.90–2001.0)	0.067^*∗∗*^

^*∗*^Chi-square test.

^*∗∗*^Mann–Whitney *U* test.

**Table 3:** AUC, *p* value, odds ratio, and prevalence of sEng, PlGF, sFlt1, IL-6, and IL-16.

	Prevalence	Gestational age		AUC	*p* value
		<34 wk^*∗*^ (%)	≥34 wk^*∗∗*^ (%)		
sEng (ng/mL)	0.439	56	44	0.610	0.106
PlGF (pg/mL)	0.446	55	45	0.684	0.005
IL-16 (pg/mL)	0.439	56	44	0.650	0.030
IL-6 (pg/mL)	0.439	56	44	0.568	NS
sFlt-1 (pg/mL)	0.439	56	44	0.511	NS
sFlt-1/PlGF	0.386	61	39	0.349	0.015

^*∗*^Early onset PE.

^*∗∗*^Late onset PE.

**Table 4:** Predictive characteristics of studied markers in relation to their cut-offs.

	Sensitivity (%)	Specificity (%)	Diag acc^*∗*^ (%)	PPV (%)	NPV (%)	LR+	LR−
sEng (ng/mL)	62.1	56.8	59.1	52.9	65.6	1.435	0.668
sFlt1 (pg/mL)	44.8	67.6	57.6	52.0	61.0	1.382	0.817
PlGF (pg/mL)	100	47.2	70.8	60.4	100	1.895	0.000
sFlt1/PlGF	96.6	5.7	46.2	40.0	100	1.022	0.621
IL-6 (pg/mL)	27.6	89.2	62.1	66.7	61.1	2.552	0.812
IL-16 (pg/mL)	69.0	67.6	68.2	62.5	73.5	2.126	0.459

^*∗*^Diagnostic accuracy (Diag acc); PPV: positive predictive value, NPV: negative predictive value, LR+: positive likelihood ratio, and LR−: negative likelihood ratio.

## Abbreviations

PE: Preeclampsia
ROC: Receiver operating characteristics
AUC: Area under the ROC curve
NA: Not applicable.
